# Genomewide transcriptional signatures of migratory flight activity in a globally invasive insect pest

**DOI:** 10.1111/mec.13362

**Published:** 2015-09-28

**Authors:** Christopher M. Jones, Alexie Papanicolaou, George K. Mironidis, John Vontas, Yihua Yang, Ka S. Lim, John G. Oakeshott, Chris Bass, Jason W. Chapman

**Affiliations:** ^1^ AgroEcologyRothamsted ResearchHarpendenHertfordshireAL5 2JQUK; ^2^ Hawkesbury Institute for the EnvironmentUniversity of Western SydneyRichmondNSW2753Australia; ^3^ Institute of Molecular Biology & BiotechnologyFoundation for Research & Technology Hellas100 N. Plastira StreetGR‐700 13Heraklion CreteGreece; ^4^ Laboratory of Pesticide ScienceDepartment of Crop ScienceAgricultural University of Athens75 Iera Odos StreetGR‐11855AthensGreece; ^5^ College of Plant ProtectionNanjing Agricultural UniversityNanjing210095China; ^6^ CSIRO Ecosystems SciencesBlack MountainClunies Ross StreetCanberraACT0200Australia; ^7^ Biological Chemistry and Crop ProtectionRothamsted ResearchHarpendenHertfordshireAL5 2JQUK; ^8^ Environment and Sustainability InstituteUniversity of ExeterPenrynCornwallTR10 9EZUK

**Keywords:** insect migration, migratory genomics, tethered flight, transcriptomics

## Abstract

Migration is a key life history strategy for many animals and requires a suite of behavioural, morphological and physiological adaptations which together form the ‘migratory syndrome’. Genetic variation has been demonstrated for many traits that make up this syndrome, but the underlying genes involved remain elusive. Recent studies investigating migration‐associated genes have focussed on sampling migratory and nonmigratory populations from different geographic locations but have seldom explored phenotypic variation in a migratory trait. Here, we use a novel combination of tethered flight and next‐generation sequencing to determine transcriptomic differences associated with flight activity in a globally invasive moth pest, the cotton bollworm *Helicoverpa armigera*. By developing a state‐of‐the‐art phenotyping platform, we show that field‐collected *H. armigera* display continuous variation in flight performance with individuals capable of flying up to 40 km during a single night. Comparative transcriptomics of flight phenotypes drove a gene expression analysis to reveal a suite of expressed candidate genes which are clearly related to physiological adaptations required for long‐distance flight. These include genes important to the mobilization of lipids as flight fuel, the development of flight muscle structure and the regulation of hormones that influence migratory physiology. We conclude that the ability to express this complex set of pathways underlines the remarkable flexibility of facultative insect migrants to respond to deteriorating conditions in the form of migratory flight and, more broadly, the results provide novel insights into the fundamental transcriptional changes required for migration in insects and other taxa.

## Introduction

The ability to initiate and sustain periods of long‐distance flight is a prerequisite for the billions of insects that migrate each year (Chapman *et al*. 2012, 2015). The behavioural, physiological and morphological adaptations necessary to undertake such flights form part of a much larger inherited ‘migratory syndrome’ which is present not only across the Insecta but also in other animal migrants such as birds and fish (Roff & Fairbairn 2007; Dingle 2014). Quantitative trait analyses in a wide range of taxa have shown that there is significant phenotypic and genetic variation in the individual traits that comprise the migratory syndrome (Pulido *et al*. 1996; Roff & Fairbairn 2007). The genes and associated biochemical pathways that underpin this variation, however, remain poorly understood.

A handful of comparative transcriptomic and genomic analyses have started to uncover differences in gene expression and single nucleotide polymorphisms (SNPs) between migratory and nonmigratory populations of the same species (Jones *et al*. 2008; Zhu *et al*. 2009; Postel *et al*. 2010; Zhan *et al*. 2014; McKinney *et al*. 2015). For example, signatures of positive selection acting upon flight muscle genes were detected in migratory populations of the monarch butterfly, *Danaus plexippus* (Zhan *et al*. 2014). Few studies have applied next‐generation sequencing to migratory insects from the same population displaying intraspecific variation in a behavioural migratory trait. Such an approach requires a clearly defined and accurate quantification of the focal trait (Liedvogel *et al*. 2011). The propensity to engage in long‐distance flight, a crucial proxy for migratory potential, can be quantified under controlled conditions using computerized tethered flight mills. Flight activity has been characterized in several insect species in this way (Colvin & Gatehouse 1993; Kent & Rankin 2001; Dorhout *et al*. 2008; Liu *et al*. 2011), but no study has yet utilized this experimental system to provide robust migratory phenotypes and then use them for downstream genotyping and gene expression analysis.

Comparative methods for determining migration‐associated genes rely on a model species that exhibits a variety of migratory phenotypes. Members of the noctuid moth family are some of the most economically damaging pests of agriculture with the capacity for long‐range movement a major contributing factor to their pest status. By far the most important species in this group is the cotton bollworm, *Helicoverpa armigera* (Lepidoptera: Noctuidae), which has an extremely large host range and is distributed globally. *H. armigera* is a facultative migrant, moving in response to deteriorating local conditions and, like other noctuid moths (Chapman *et al*. 2008, 2010), they utilize favourable high‐altitude wind currents to optimize their movement direction and ground speed (Feng *et al*. 2009). Evidence for these movements is supported from entomological radar, insect trapping and geneflow studies (Zhou *et al*. 2000; Scott *et al*. 2005; Feng *et al*. 2009). Not all populations of *H. armigera* are migratory, however, and the migratory syndrome may only be expressed in certain years depending on the local conditions (Scott *et al*. 2005). This plasticity makes *H. armigera* an attractive species for studying the genetic basis of migration as a variety of phenotypes is likely to persist in natural populations.

To dissect the molecular basis of flight in a migratory insect, we focus here on the propensity for long‐distance flight and use an unique integrative approach of tethered flight and transcriptomics to (i) quantify the intraspecific variation in flight activity during the migratory phase of recently collected field populations of *H. armigera* from different geographic origins, (ii) identify the differentially expressed suite of genes between flight phenotypes that contribute to the migratory capacity of *H. armigera* and (iii) validate the expression of these genes in independently flown *H. armigera* adults.

## Materials and methods

### *H. armigera* collections

The adult *H. armigera* used in the flight mill, RNA‐seq and qPCR experiments were collected from Bt cotton from five populations in China [Dafeng (Jiangsu province), Anyang (Henan province), Jingzhou, (Hubei province), Qiuxian (Hebei province) and Wanjiang (Anhui province)] and a single site in northern Greece during the summer of 2013 and shipped to Rothamsted Research, UK (Table S1, Supporting information). Eggs were collected from the field populations of Dafeng and Jingzhou and reared for a further two generations. Anyang and Qiuxian adults were collected in light traps and reared for one subsequent generation in the laboratory. *H. armigera* from China were considered separate ‘populations’ for the study although gene flow between these areas may exist given the long‐distance migratory ability of *H. armigera* in China (Feng *et al*. 2009). *H. armigera* from cotton fields in northern Greece (41°N, 023°E) were collected as 4th‐5th larval instars and reared for one generation in the laboratory. An additional long‐term laboratory strain, Bayer (courtesy of the Max Planck Institute, Jena, Germany), was used in the qPCR experiments. Insects were reared under a constant light regime of L:D 14:10 at 26 ± 1 °C, and the flight mill experiments conducted under the same conditions. Larvae were reared individually in 37‐ml clear plastic polypots containing a wheat germ artificial diet (Vanderzant *et al*. 1962).

### Tethered flight mills

**Figure 1:**
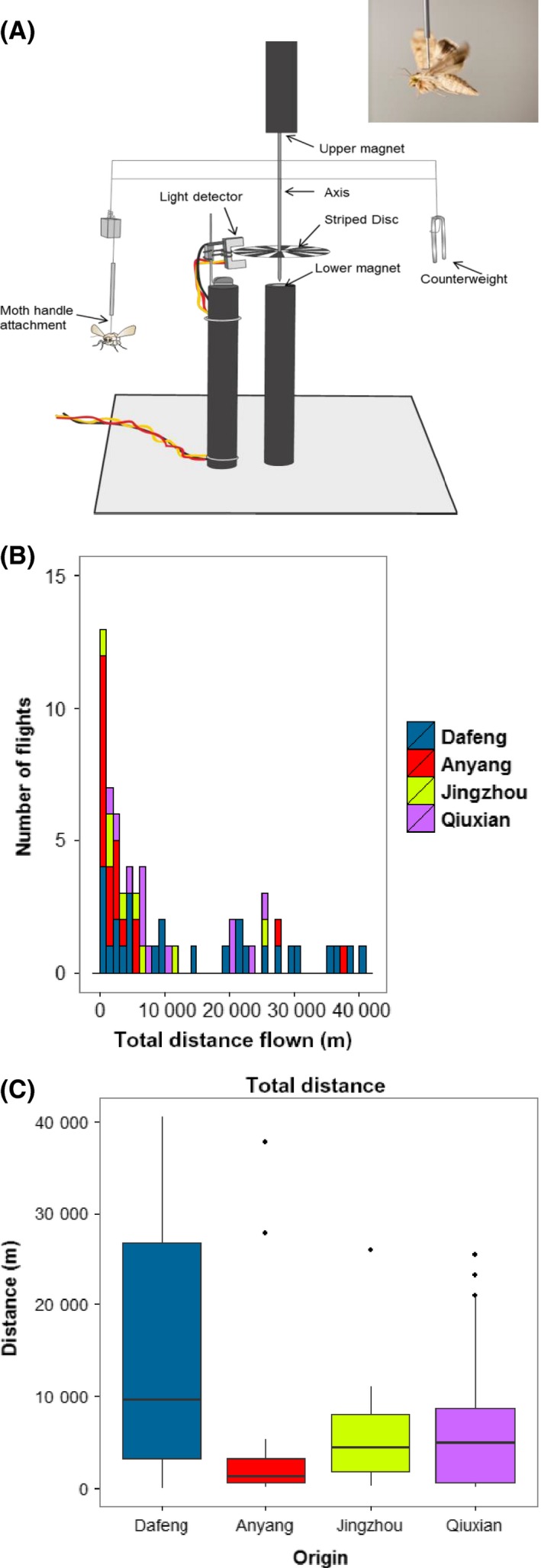
Variation in flight activity of adult *H. armigera* from China characterized using tethered flight. (A) Diagram of tethered flight mill system for quantifying flight behaviour in noctuid moths. Upper panel shows single *H. armigera* female attached to the handle ‘in‐flight’. (B) Variation in the total distance flown by the four Chinese populations. Each bin represents 1000 metres flown during the course of a single night. The origin of each insect is given according to the colour code highlighted. (C) Box plots of the total distance flown by the four populations from China (Dafeng, *n *=* *27; Anyang, *n *=* *19; Jingzhou, *n *=* *8; Qiuxian, *n *=* *19).

Flight experiments were conducted in October and November 2013 using newly built computerized tethered flight mills designed and housed at Rothamsted Research (Fig. 1A). In brief, the flight arm is made from twisted wire with the vertical axis secured between two magnets. The insect is attached to a pin that fits into a sleeve suspended from the flight arm. This allows the insect to fly rotationally in a horizontal plane. A sensor detects a rotational disc so that the speed and time of the rotation (and hence the powered flight of the insect) are recorded. The time period between insect collection and quantifying flight activity (3 months) ensured that we captured natural flight behaviour in the shortest time possible. Up to 16 insects were flown during a single night. Each morning pupae were checked for emergence, and any adults were put aside for flight mill experiments. Any moths with damaged wings or unhealthy were not chosen for experiments. All moths were flown the night post‐eclosion (1 day old) to capture prereproductive behaviour (Colvin & Gatehouse 1993). Experimental insects were sedated at 4 °C for a minimum of 2 h prior to tethering. Scales were removed from the thorax and a small amount of adhesive glue applied to both a *c*.60‐mg pin and the exposed thorax. Prior to the flight, each insect was provided with a *c*.20% honey solution ad libitum. All insects were randomly assigned to a flight mill before 1900 each evening. A paper platform was provided to allow the moth to rest prior to undertaking its initial flight. Data were collected between 1900 and 0830 with the 10‐h dark/night cycle running between 2000 and 0600. At 0900, the following day, the insects were taken off the flight mills and allowed to recover for 1–2 h before being snap‐frozen in liquid nitrogen and stored at −80 °C until RNA extraction. Any insects which looked damaged, unhealthy or had escaped from their pin were disregarded from further analyses. In an initial experiment, male and female adult *H. armigera* from four Chinese populations (Dafeng, Jingzhou, Qiuxian and Anyang) were flown simultaneously over the course of a single week to determine intraspecific population differences. Insects from Wangjiang, Greece and Bayer were flown in independent experiments.

### REML analysis of total distance flown

To explore the relationship between the total distance flown by Chinese *H. armigera* and the explanatory variables, *population* and *sex*, we computed variance components using a restricted maximum likelihood (REML) approach. *Population* and *sex* were considered fixed effects, while the night the insect was flown (*night*) and the mill used (*mill*) were treated as random effects. Using the initial (full) fixed model, the individual random components were sequentially dropped from the model and the deviance between subsequent ‘simpler’ models compared. After determining the variance model, the fixed terms were sequentially dropped to provide the explanatory variables that were associated with the total distance flown (*F* statistic, *P *<* *0.05). Following the observation that insects from Dafeng flew the furthest distance on the flight mills and nonoverlapping confidence intervals from the model predictions (Fig. S1, Supporting infromation), a similar REML approach was used to determine whether insects from Dafeng flew significantly further than the three other populations. Total distance flown was squared‐root‐transformed prior to the statistical analysis. The outputs from the full REML models are provided in Table S2 and Table S3 (Supporting information). All analyses were conducted in genstat 16th edition.

### RNA extraction and sequencing

Total RNA was extracted from three whole individual adult females per sample using the ISOLATE II RNA Mini Kit (Bioline). Each flight phenotype consisted of three biological samples (nine insects per phenotype) which were chosen prior to extraction according to the total distance flown. RNA was sent to The Genome Analysis Centre (TGAC, BBSRC, Norwich, UK) for library construction (Illumina TrueSeq Library construction) and sequencing.

### Mapping RNA‐seq reads to *H. armigera* genome

Paired‐end (PE) read 100‐bp sequencing was performed on six samples from each experiment on half a flow cell of the Illumina HiSeq 2500. This yielded *c*.14.4–27.8 million PE reads per sample. Alignment, correction of illumina and isoform bias, FPKM estimation and TMM normalization were performed using DEW (http://dew.sourceforge.net/). Reads from each sample were mapped to the genes of the *H. armigera* genome to find those transcripts with ≥95% amino acid identity and no more than 10% length difference (CD‐HIT –s 0.90 –c 0.95). The raw reads were then realigned to these transcripts using GSNAP (Wu & Nacu 2010) and the alignment edited to correct for illumina bias and multi‐isoforms using eXpress (Roberts & Pachter 2012). A total of 13 776 transcripts had at least 4 mapped reads in at least one sample (80.97%). The number of reads mapping to Greece and China samples was 12,255 (72.09%) and 13,289 (78.17%), respectively.

### Differential expression

To detect differential gene expression between flight phenotypes, we used two expression packages edgeR and DEseq2 (Anders *et al*. 2013). Both packages use the negative binomial model for analysing RNA‐seq count data but differ in their estimation of gene dispersal. Using both methods, we aimed to capture as many differential transcripts as possible. The China and Greece data sets were analysed separately and all genes that were statistically significant at a false discovery rate (FDR) <0.1 were considered candidates for flight performance. For edgeR, any transcript with less than five reads for all samples were removed from the analysis. All analyses were performed in r software (R Core Development Team 2014). An enrichment analysis (Fisher's exact test) of GO terms for differentially expressed transcripts (upregulated and downregulated genes tested separately) was performed against the reference gene set in Blast2GO at an FDR < 0.05 (Götz *et al*. 2008).

### Quantitative PCR

A subset of differentially expressed genes (*N *=* *16) were tested using qPCR to validate the RNA‐Seq data and to independently test expression in additional *H. armigera* flown on the flight mills. The same samples from RNA‐seq were used for Dafeng, Anyang and Greece. For the additional samples, total RNA was extracted from flight phenotypes from Wangjiang and Bayer as described above. Total RNA (600 ng) was converted into cDNA using oligo(dT)_20_ (Invitrogen) and SuperScript III (Invitrogen). Exon–exon spanning primers for target genes were designed using Primer‐BLAST (NCBI) (Ye *et al*. 2012). Two previously designed control genes, *elongation factor 1‐*α and β*‐actin* (Wang *et al*. 2013; Yan *et al*. 2013), were used for internal normalization. The specificity, dynamic range and PCR efficiency of each primer set were determined by testing against a fivefold serial dilution of cDNA (from 1/10th to 1/6000th). Details of all primers are given in Table S4 (Supporting information). All PCRs (20 μl) were run on the RotorGene 6000 (Qiagen) using 300 nm of each primer, 10 μl SYBR Green JumpStart *Taq* ReadyMix (Sigma Aldrich) and 5 μl of cDNA diluted 50‐fold. PCR conditions used throughout were 95 °C for 2 min followed by 40 cycles of 95 °C for 10 s, 57 °C for 15 s and 72 °C for 10s. Melt curves were run after amplification to check for specificity. Reactions were run in duplicate with no template controls (distilled water) for each primer set. Data were preprocessed to remove outliers (SD of technical replicates > 0.5) and late Ct values (>35). Expression levels were calculated according to the ddCt method following correction for PCR efficiency and normalization for the two control genes (Schmittgen & Livak 2008). qPCR experiments followed best practices according to the MIQE guidelines (Bustin *et al*. 2009).

## Results and discussion

### Continuous variation in flight activity of *H. armigera*

The nocturnal flight activity of *H. armigera* was characterized using a newly developed state‐of‐the‐art tethered flight mill system (UK Patent Application No. 1314415.9; Fig. 1A). Each insect was flown for a single night (from 19:00 to 08:30 GMT) in a controlled environment with the speed, distance and duration of all flights electronically recorded. In the first series of experiments, adult moths originating from four field populations from across China were flown shortly after collection (*N *=* *73; Table S1, Supporting information). Assessment of the total distance flown indicated there was no delineation between short‐ and long‐distance fliers but rather continuous variation (Fig. 1B), similar to other flight mill studies (Colvin & Gatehouse 1993; Schumacher *et al*. 1997). The maximum distance covered by a single individual during one night was 40.6 km. Restricted maximum likelihood (REML) modelling explored the association of population and sex on the total distance using the mill and night flown as random effect variables. There was strong evidence for population differences for the distance flown (*F*_3,61_ = 4.73, *P *=* *0.005) with no evidence that the sex of the insect had any effect (*F*_1,32_ **=* *0.48*, P *=* *0.492; Table S2, Supporting information). Of the four Chinese populations flown, adult moths from Dafeng flew the furthest mean distance (mean distance* *=* *15,430 m; Fig. 1C) and their total distance was significantly greater than the three other populations (*F*_1,57_ **=* *13.47, *P *= <0.001; Table S3, Supporting information) consistent with predictions from the REML and nonoverlapping 95% confidence intervals (Fig. S1, Supporting information). We also wanted to capture the degree of variation in flight activity within a single *H. armigera* population using a single sex, and for this, we used female insects originating from northern Greece (*N *=* *28). The mean distance covered by females from Greece was 17 350 m ranging from 457.8 m to 35 430 m (Fig. S2, Supporting information).

All *H. armigera* adults were flown during the prereproductive period (PRP) (the night following eclosion) when migratory flight activity is known to occur in this species (Colvin & Gatehouse 1993). Seasonal migrations are reported across central and northern China (Feng *et al*. 2009), while in northern Greece, there is evidence for the existence of a local overwintering population supplemented with immigrants in the spring, possibly from North Africa (Mironidis *et al*. 2010). On this basis, it is likely that the *H. armigera* used in our experiments possess, to at least some degree, the genetic adaptations necessary for migratory flight. Heritability estimates indicate a strong genetic component for flight activity in prereproductive *H. armigera* (Colvin & Gatehouse 1993), and the variation in the propensity for long‐distance flight observed both within and between populations (Fig. 1B and Fig. S2, Supporting information) suggests that fundamental physiological and behavioural differences underlie the flight potential of insects used in this study.

### Genomewide transcription profiles of *H. armigera* flight phenotypes

**Figure 2:**
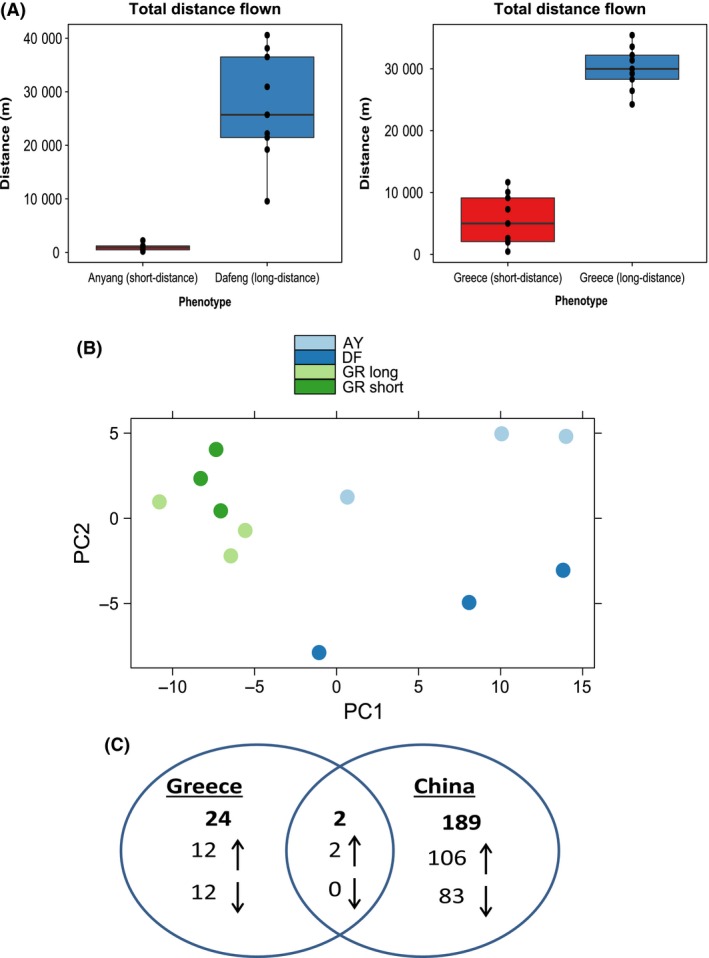
RNA‐seq of *H. armigera* flight phenotypes from China and Greece. (A) Boxplots of total distance flown by moths used in the RNA‐seq comparisons between short‐ and long‐distance fliers of *H. armigera* from Greece and China. The magnitude of difference between short‐ and long‐distance fliers is much greater in China than Greece (30.6‐fold vs. 4.1‐fold). (B) PCA plot of log_2_‐transformed read counts for each sample used in RNA‐seq. (C) Venn diagram showing the number of differentially expressed genes between flight phenotypes from Greece and China. All genes were significant at FDR < 0.1 using the edgeR and/or DESeq2 package.

Next, we compared genomewide transcription profiles in short‐ and long‐distance fliers characterized using the flight mills. Two separate RNA‐seq analyses were performed both within and between *H. armigera* populations. The within‐population comparison was performed on Greek moths, while the between‐population comparison was conducted on Dafeng and Anyang from China. To capture true differences in flight behaviour, RNA was extracted from whole insects that represent the opposite ends of the flight mill data distribution and were categorized as short‐ and long‐distance phenotypes (Fig. 2A). Total RNA was subject to Illumina cDNA library preparation and sequencing. A total of *c*.286 million paired‐end 100‐bp reads were sequenced with an average yield of 14.4–26.8 million reads per sample.

Following quality control, reads were aligned to the annotated *H. armigera* genome, isoform variation was accounted for and a combination of FPKM and TMM normalization using Differential Expression on the Web (DEW) (http://dew.sourceforge.net) produced robust gene expression estimates. PCA showed distinct transcriptional signatures for each flight phenotype (Fig. 2B). Differential expression between flight phenotypes was determined at a false discovery rate (FDR) of *P *<* *0. 1 using a union of two separate methods as implemented in edgeR and DESeq2 (Anders *et al*. 2013). Any gene significantly expressed in either the China or Greece experiment was considered a candidate gene for flight. Using this approach, we identified 215 differentially expressed genes from the estimated 17 001 genes in the *H. armigera* genome (1.26%). (Fig. 2C; Data S1, Supporting information). Of the 215 candidates, the majority come from the analysis between short‐ and long‐distance fliers from China (*N *=* *191) compared to the intrapopulation study from Greece (*N *=* *26). This could reflect the geographical separation between Dafeng and Anyang (*c*. 650 km) or the greater contrast in flight activity between the two phenotypes (Fig. 2A). There was a balance in the share of upregulated and downregulated genes (up* *=* *55.8%; down* *=* *44.2%), and of the two common genes expressed in both data sets (OBP6 and vanin‐like protein 3), the fold‐change direction was consistent. To validate our expression data, qPCR was used to measure the expression of a subset of genes using the same RNA samples. There was a good agreement between the RNA‐seq and qPCR data with a stronger correlation between fold‐changes observed for DESeq2 (*R* ^2^ **=* *0.69) (Fig. S3, Supporting information) as reported elsewhere (Anders *et al*. 2013).

### Expression patterns reflect metabolic demands of prolonged flight in *H. armigera*

**Figure 3:**
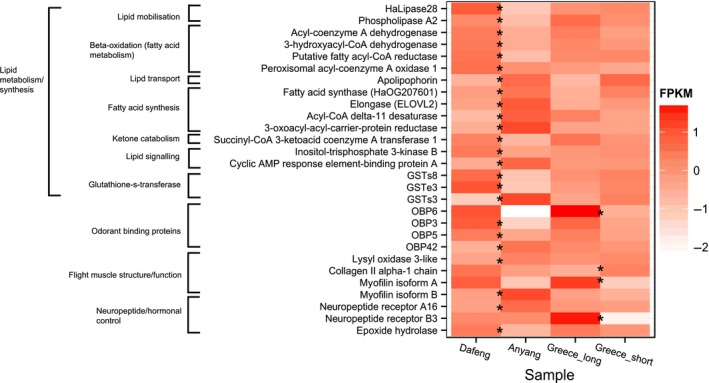
Differential expression of core genes with an identified role in flight physiology. Heat map of absolute expression measured as normalized FPKM values. Values are given for all genes with differential expression between the flight phenotype comparisons (FDR < 0.1) represented by *. The two flight phenotypes from Greece are marked as ‘Greece long’ and ‘Greece short’.

As evidenced by the functional annotation (Fig. 3), a striking proportion of the 215 differentially expressed genes are directly associated with flight physiology. The evolution of prolonged flight in migratory insects, as well as in birds, has led to substantial physiological adaptations including the efficient mobilization of energy reserves, morphological changes for flight mechanics, coordinated hormonal control and the ability to cope with the stresses imposed by extreme energetic demands. The transcriptional profiles of several genes expressed between the *H. armigera* flight phenotypes reflect these adaptations and are discussed below.

Insect flight is the most energy demanding activity known in the animal kingdom requiring metabolic rate increases of 50‐ to 100‐fold (Arrese & Soulages 2010). The expression data are consistent with the extreme metabolic loads placed on migratory insects during flight. First, we observed several functional GO terms in an enrichment analysis of overexpressed genes in long‐distance fliers related to energy production including the purine nucleotide and inosine monophosphate (IMP) pathways (Table S5, Supporting information). Both of these pathways are essential for ATP turnover which can increase several hundred‐fold in working insect flight muscles (Candy *et al*. 1997). Increases in AMP and IMP have been recorded in locust flight muscle (Weyel & Wegener 1996), and the majority of upregulated transcripts in a microarray comparison of winged versus unwinged pea aphids (*Acyrthosiphon pisum*) were involved in energy production (Brisson *et al*. 2007).

**Figure 4:**
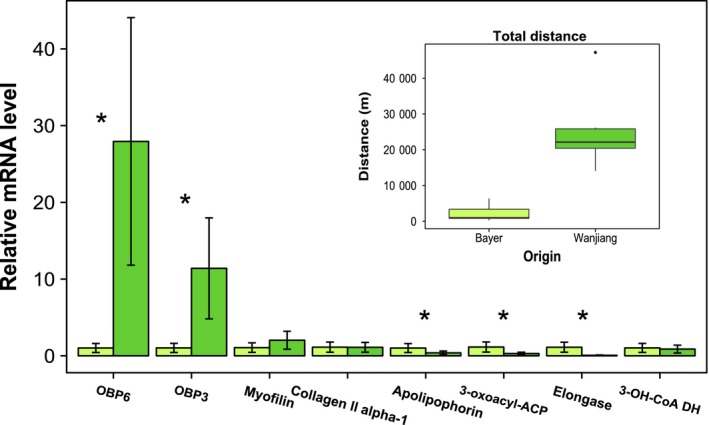
Transcription of candidate genes for flight in an independent sample set. Expression levels of eight genes identified from RNA‐seq were tested using qPCR in *H. armigera* flown in an independent flight mill study. Boxplots (inset) show the total distance flown of a subsample of field‐collected adult females from Wanjiang (China) and a laboratory strain (Bayer). The eight genes tested were picked to cover a range of physiological processes contributing to flight (odorant binding proteins (OBP3 and OBP6), flight muscle structure (myofilin and collagen II α‐1), fatty acid synthesis (3‐oxoacyl‐ACP‐reductase, elongase), fatty acid metabolism (3‐hydroxyacyl‐CoA dehydrogenase) and lipid transport (apolipophorin)). qPCR values are relative to the average of the three biological replicates from Bayer, and significant expression is denoted by * at *P *<* *0.05 (*t*‐test of log_2_‐transformed values).

Migratory insects such as locusts and noctuids need to power flight muscle activity for extended periods and do so mainly via the aerobic breakdown of high‐energy lipids in the form of triacylglycerol (TAG). The metabolism of fatty acids (FA) in the mitochondria of insect flight muscle cells occurs through β‐oxidation. The enzymes that catalyse the first and third reaction of the β‐oxidation pathway (fatty acyl‐CoA dehydrogenase and 3‐hydroxyacyl‐CoA dehydrogenase) were upregulated in Dafeng compared to Anyang (1.81–1.83‐fold) (Fig. 3). A copy number expansion of genes encoding β‐oxidation enzymes was described in the migratory locust (*Locusta migratoria*) providing some evidence that adaptations in this pathway contribute to long‐distance flight capacity (Wang *et al*. 2014). β‐Oxidation also occurs in peroxisomes and is required to oxidize very long‐chain fatty acids that cannot be broken down by mitochondria (Wanders *et al*. 2001). The first step of this reaction is catalysed by peroxisomal acyl‐CoA oxidase which was significantly upregulated in Dafeng moths (*c*.1.4‐fold). Transcriptional regulation of lipid metabolism is supported by the concomitant downregulation of enzymes catalysing the reverse process, fatty acid synthesis (Fig. 3). These include fatty acid synthase, 3‐oxoacyl‐ACP‐reductase, acyl‐coA δ desaturase and an elongase (*ELOVL2*). 3‐oxoacyl‐ACP‐reductase and elongase were significantly downregulated in an independent qPCR assessment of additional *H. armigera* flight phenotypes (Fig. 4). Inositol‐triphosphate kinase is associated with the inactivation of the first catalytic step of FA synthesis and was also upregulated in Dafeng moths.

Further evidence for lipid‐fuelled flight is the expression of two lipases (HaLipase28 and phospholipase A2) and an isoform of the lipid transport protein apolipophorin. Lipases are essential for mobilizing lipids from TAG stores, and the insect adipose triglyceride lipase, an important enzyme in *Drosophila* metabolism, is a member of the phospholipase A2 family (Arrese & Soulages 2010). Insect flight muscles do not directly store lipids and require an efficient system to transport hydrophobic fatty acids to the muscle cell which in locusts is mediated by apolipoproteins (Haunerland 1997). Finally, there was evidence that *H. armigera* use ketone bodies as supplementary fuel for flight with the upregulation of succinyl‐CoA: 3‐ketoacid coenzyme A transferase 1 (*SCOT*), a key enzyme for mediating energy production from ketone. Ketone bodies are present in both the fatty tissues and flight muscles of moths and locusts and have been implicated as an important substrate for flight fuel (Candy *et al*. 1997).

A negative outcome of high ATP turnover and lipid‐fuelled flight activity is oxidative damage (Magwere *et al*. 2006). The expression of gene families possessing antioxidant properties, such as glutathione S‐transferases (GSTs), protects insects from oxidative stress. Three GSTs were among our expressed data set two of which are members of the sigma class of GSTs and are isoform orthologues of *Drosophila melanogaster GSTs1* (Fig. 3). *GSTs1* is highly abundant in *D. melanogaster* indirect flight muscle and protects against 4‐hydroxynonenal (4‐HNE), a derivative of lipid peroxidation (Singh *et al*. 2001). The expression of sigma GSTs in the insect fat body and copy number expansion in *L. migratoria* (Wang *et al*. 2014), coupled with our data presented here, suggests that *H. armigera* may have evolved a similar adaptation to coping with oxidative stress from prolonged flight.

Odorant binding proteins (OBPs) are small extracellular transporter proteins (13–16 kDa) which capture and transport hydrophobic ligands (e.g. pheromones) (Zhou 2010). Our data set contained four OBPs, one of which, OBP6, was significantly upregulated in moths displaying long‐distance phenotypes from both China and Greece (Fig. 3). OBP6 and OBP3 were two of the most highly expressed genes in long‐distance moths from China (Fig. 3) confirmed in the independent assessment of additional flight phenotypes (Fig. 4). While many insect OBPs are antennal specific, both OBP6 and OBP3 are expressed in the insect body (Gu *et al*. 2014) suggesting a role other than olfaction. All moths were flown in the prereproductive period (<1 day old) with no access to host plants so it is highly unlikely that the expression of OBPs represents host‐seeking or mating behaviour. The hydrophobic binding capacity of OBPs suggests that this group of proteins may act as lipid transport proteins in *H. armigera*.

### Flight activity associated with genes encoding flight muscle proteins

We identified differentially expressed genes strongly associated with flight muscle structure. The α1‐subunit of collagen type II was downregulated in long‐distance flying moths from Greece (3.10‐fold). Whole‐genome sequencing of migratory and nonmigratory monarch butterflies (*D. plexippus*) revealed positive selection surrounding the collagen type IV, subunit α1, in migratory North American populations (Zhan *et al*. 2014). Collagen IV forms the central component of basement membranes essential for muscle structure and function and the downregulation of this gene in migratory monarchs, coupled with decreased metabolic rates during flight, suggested the evolution of greater metabolic efficiency in the migratory forms (Zhan *et al*. 2014). Collagen II is the main structural component of cartilage in humans (Garofalo *et al*. 1993) but virtually no information is available on the role of this protein in insects; however, it is possible the downregulation of this gene represents a similar adaptation for greater flight efficiency. The concomitant downregulation of lysyl oxidase supports this finding as this enzyme is essential for maintaining the integrity of collagen structure (Li *et al*. 1997). The expressed gene set also included two isoforms of myofilin, a 20‐kDa protein associated with myosin in the core of thick filaments that make up asynchronous insect flight muscle (Qiu *et al*. 2005). The transcription of genes coding for proteins associated with locomotory muscles (e.g. tropomyosin) has been described in long‐winged forms of the cricket *Gryllus firmus* (Vellichirammal *et al*. 2014) as well as other migratory organisms (Postel *et al*. 2010).

### Evidence for hormonal control of migratory flight activity

Juvenile hormone (JH) plays a central role in the trade‐off between reproductive and migratory physiology in insects with lower JH titres favouring migration (Roff & Fairbairn 2007). The *Bombyx mori* homologue of the neuropeptide receptor A16 (*BNGR‐A16*), downregulated in long‐distance moths from China (FC* *=* *2.67), is the receptor for the hormone allatotropin (AT) which stimulates juvenile hormone (JH) biosynthesis (Yamanaka *et al*. 2008). The relationship between AT mRNA expression and JH levels is tightly coupled and injection of AT induces resident behaviour in migrant insects (McNeil *et al*. 2005; Zhu *et al*. 2009; Jiang *et al*. 2011). The downregulation of the AT receptor, *BNGR‐A16*, in long fliers from Dafeng suggests that the contrasting flight propensities between Dafeng and Anyang (Fig. 1B) are a reflection of true differences in migratory potential. The upregulation of an enzyme with JH degrading properties, epoxide hydrolase, supports evidence for the regulation of JH levels in insects from this population.

### Summary – a complex suite of genes underlies migratory flight activity in *H. armigera*

Migration biology is set to enter the genomic era as the number of sequenced genomes from nonmodel migratory species increases (Liedvogel *et al*. 2011). While there is evidence of selection acting on genes involved in a single trait associated with migration in insects (e.g. flight muscle proteins in the monarch butterfly (Zhan *et al*. 2014), the migratory syndrome is polygenic and the consequence of a complex set of genes and the interactions between them (Roff & Fairbairn 2007). By combining accurate phenotypic quantification of a migratory trait and next‐generation sequencing, we hypothesize that the genes expressed in *H. armigera* displaying contrasting flight activities (and hence different migratory potentials) are strong candidates driving this essential life history characteristic. This complex suite of genes encode proteins for a broad range of physiological functions, and while many have clear direct associations with flight capacity, it is likely that the expression of a subset of our candidate genes is consistent with other aspects of migratory biology and additional pleiotropic effects. The functional role of each gene on migratory behaviour in this species and other noctuid moths needs further investigation. The activity of these proteins will undoubtedly differ between species and experimental approaches; however, they provide a valuable insight into the genetic adaptations that drive this fascinating phenomenon for future comparative studies in insects and other migratory taxa, including birds. It is worth noting that the propensity to engage in migratory flight is intrinsically linked to the physiological state of many insects with the PRP a key window within which migratory behaviour occurs (Dingle 2014). The duration of PRP varies in *H. armigera* reflecting differences in migratory potential between individuals or populations (Colvin & Gatehouse 1993). The transcription of genes associated with migratory flight will undoubtedly vary during the PRP (e.g. genes associated with juvenile hormone; Jiang *et al*. 2011). To reliably assess transcriptional differences between flight phenotypes, it is necessary to sample insects at a comparable age (1 day posteclosion in the present study); however, time‐course analyses of migratory insects during the ‘migratory window’ may capture additional optimal gene expression profiles that contribute to an insect's full migratory capacity. Finally, facultative migrants, such as noctuid moths, must possess the genetic architecture to migrate and fly substantial distances within a single generation in response to adverse prevailing conditions. We propose that the ability to switch on and express many of the genes identified in this study allow *H. armigera* to power flight to high altitudes for extended periods of time and contribute to its status as a global pest.

C.M.J., K.S.L., C.B. and J.W.C. conceived and designed the study. Y.Y., J.V. and G.K.M. performed field sampling and provided insect material. C.M.J. performed all tethered flight mill experiments and molecular laboratory work. A.P. and J.G.O. provided genomic resources. C.M.J. and A.P. conducted RNA‐seq analyses. C.M.J., A.P., J.G.O., C.B. and J.W.C. wrote the manuscript.

## Data accessibility

The flight mill data, raw RNA‐seq read count data, output from edgeR and DEseq2, GO terms and raw qPCR data (Ct and validation analysis values) are deposited in the online Dryad Digital Repository (www.datadryad.org; doi:10.5061/dryad.bp7j7). RNA‐seq fastq files and experimental information are available at ArrayExpress (Accession no. E‐MTAB‐3790). Gene expression data for candidate genes are provided as supporting information in file: Jones_et_al_DataS1.xlsx.

## Supporting information


**Figure S1.** REML estimations of total distance flown by Chinese populations.
**Figure S2.** Flight performance of *H. armigera* from northern Greece.
**Figure S3.** Validation of RNA‐seq by qPCR.
**Table S1.** Information on the origin and generation of each *H. armigera* collection used in either flight mill, RNA‐seq and/or qPCR experiments.
**Table S2.** Output of explanatory variables from full REML model for total distance flown by Chinese *H. armigera*.
**Table S3**. Output of explanatory variables from full REML model investigating total distance flown by Dafeng *H. armigera* versus three other Chinese populations.
**Table S4.** Primer information for target and control genes used to validate and analyse expression in *H. armigera*.
**Table S5.** GO‐term enrichment analysis of genes up‐regulated in long‐distance phenotypes of *H. armigera*.

**Data S1.** A list of differentially expressed genes, corresponding annotations and fold changes for the China and Greece flight mill experiments.
