# Supplementary material for: Genomewide transcriptional signatures of migratory flight activity in a globally invasive insect pest
Source: Mol Ecol. 2015 Sep 28;24(19):4901–11. doi: 10.1111/mec.13362 (PMC5102652; doi:10.1111/mec.13362)
Supplement: Supplementary file 1 — Figure S1. REML estimations of total distance flown by Chinese populations. Figure S2. Flight performance of H. armigera from northern Greece. Figure S3. Validation of RNA‐seq by qPCR. Table S1. Information on the origin and generation of each H. armigera collection used in either flight mill, RNA‐seq and/or qPCR experiments. Table S2. Output of explanatory variables from full REML model for total distance flown by Chinese H. armigera. Table S3. Output of explanatory variables from full REML model investigating total distance flown by Dafeng H. armigera versus three other Chinese populations. Table S4. Primer information for target and control genes used to validate and analyse expression in H. armigera. Table S5. GO‐term enrichment analysis of genes up‐regulated in long‐distance phenotypes of H. armigera. [file MEC-24-4901-s001.docx]

**SUPPORTING INFORMATION**

**Genome-wide transcriptional signatures of migratory flight activity in a globally invasive insect pest**

**Christopher M. Jones, Alexie Papanicolaou, George K. Mironidis, John Vontas, Yihua Yang, Ka S. Lim, John G. Oakeshott, Chris Bass and Jason W. Chapman**


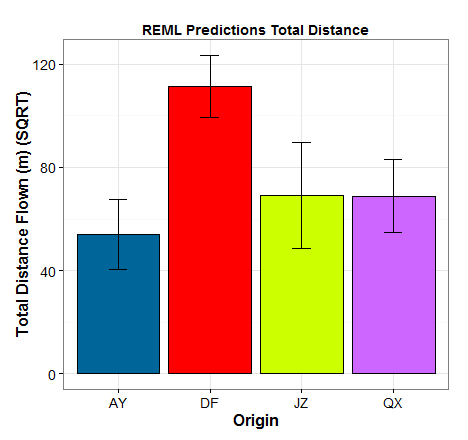


**Figure S1.** REML estimations of total distance flown by Chinese populations. Total distance flown is square root transformed and error bars are 95% CIs. Adult moths from Dafeng flew significantly further than the other three populations (*P <* 0.001) supported by non-overlapping 95% CIs.


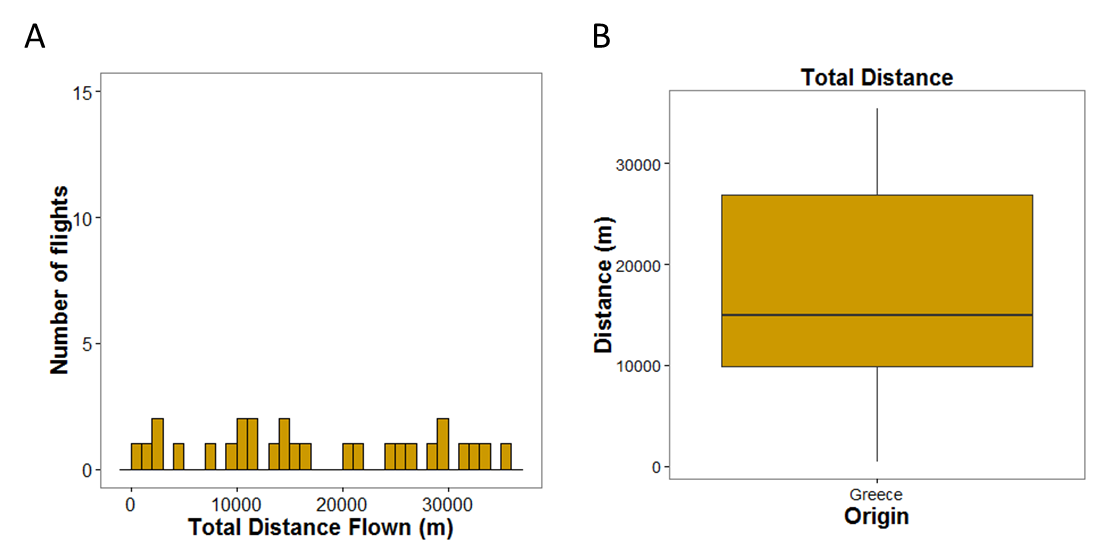


**Figure S2.** Flight performance of *H. armigera* from northern Greece. The (A) distribution and (B) boxplot of total distance flown are shown (*n* = 28). Only female moths from the Greek population were flown.


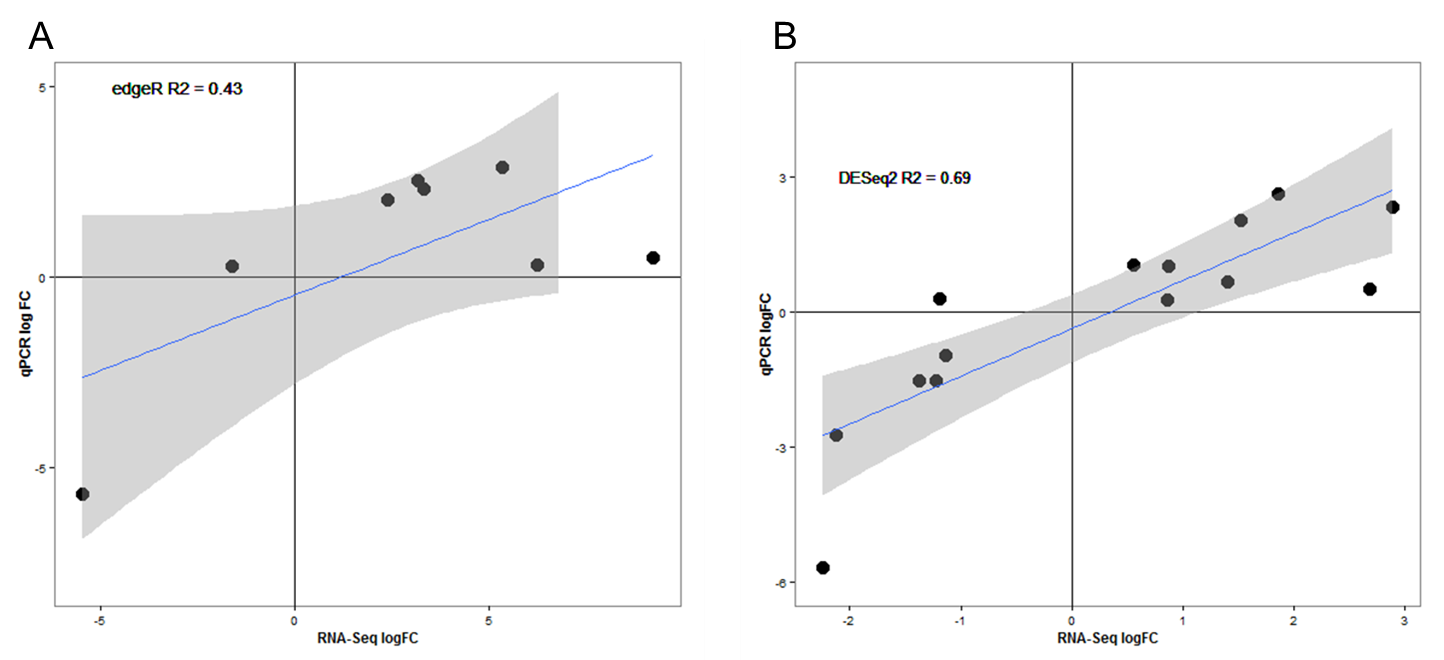


**Figure S3.** Validation of RNA-seq by qPCR. Individual qPCR fold-changes are plotted against RNA-seq values per expression package (A) edgeR and (B) DESeq2. Only instances in which the transcript was significantly expressed in the RNA-seq data (FDR < 0.1) are plotted. Fold-changes are log_2_-transformed. A simple linear regression line is presented (with 95% CIs) with the corresponding R^2^ value for each package.

**Table S1.** Information on the origin and generation of each *H. armigera* collection used in either flight mill, RNA-seq and/or qPCR experiments.

| Country of origin | Population | Reference coordinates |
| --- | --- | --- |
| China | Dafeng | 33.19°N, 120.50°E |
| China | Anyang | 36.10°N, 114.20°E |
| China | Jingzhou | 30.14°N, 111.73°E |
| China | Qiuxian | 36.81°N, 115.16°E |
| China | Wangjiang | 30.14°N, 116.66°E |
| Greece | Greece | 41°N, 023°E |
| Laboratory | Bayer | - |

**Table S2.** Output of explanatory variables from full REML model for total distance flown by Chinese *H. armigera*.

| Fixed term | F statistic | n.d.f | d.d.f | F-test *P* |
| --- | --- | --- | --- | --- |
| population | 4.73 | 3 | 60.6 | 0.005 |
| sex | 0.48 | 1 | 32.4 | 0.492 |
| population*sex | 0.27 | 3 | 63.4 | 0.844 |

Constant + population + sex + population*sex (night + mill + night*mill)

**Table S3**. Output of explanatory variables from full REML model investigating total distance flown by Dafeng *H. armigera* versus three other Chinese populations.

| Fixed term | F statistic | n.d.f | d.d.f | *F*-test *P* |
| --- | --- | --- | --- | --- |
| dafeng | 13.47 | 1 | 56.9 | 13.47 |
| dafeng*rest | 0.38 | 2 | 61.6 | 0.38 |
| sex | 0.48 | 1 | 32.4 | 0.48 |
| dafeng*sex | 0.63 | 1 | 64.6 | 0.63 |
| dafeng*rest*sex | 0.09 | 2 | 63.3 | 0.09 |

Constant + dafeng + dafeng*rest +sex +dafeng*sex + dafeng*rest*sex (night + mill + night*mill)

**Table S4.** Primer information for target and control genes used to validate and analyse expression in *H. armigera*.

| Type | Gene description | gene name | Primer Fwd (5'-3') | Primer Rev (5'-3') | Efficiency | |
| --- | --- | --- | --- | --- | --- | --- |
| target | obp6 | HaOG200803 | TGAGCAAAGTTCACGCCACA | GGGTAACTCCGTGCTCCTTA | 0.93 |  |
| target | obp3 | HaOG200802 | GACCAAGCTGCAGAAGTTCG | ATTCACTGCAGCGCATGTCT | 0.79 |  |
| target | BMORI:retinoid-inducible serine carboxypeptidase-like isoform X1 | HaOG202884 | TTCAAAGCTGGACCCAGACG | CTGACTGTCCGGCAGCATTA | 0.81 |  |
| target | BMORI:nucleoside diphosphate kinase 7-like | HaOG216795 | TGAAGATGCGCGAATGATGC | CCGTGAGAGTTGAGCGGAAT | 1.08 |  |
| target | BMORI:neuropeptide receptor B3 precursor | HaOG206273 | CGATACCAGCCAGTGTGAGG | AGTGATGACAAGAAGGCCCG | 0.76 |  |
| target | BMORI:myofilin isoform A | HaOG212567 | ATCATACTGTCTTCCCTCAACTTTT | GAAGCCCCTGCTGTTATTCAA | 0.79 |  |
| target | HaLipase28 | HaOG200555 | CATAACGCGACCATGATGCC | CCTTGACCGTAGTGAGCGAG | 1.06 |  |
| target | BMORI:collagen alpha-1(II) chain-like | HaOG215487 | ATATCCGGTCCAGGTGGGTC | TAGGGTTCAATCCGTTACCGC | 0.83 |  |
| target | BMORI:apolipophorins isoform X2 | HaOG211110 | GTCAGGATCTTGCGTGCCT | AGACCTCAATGCTGTAGCCG | 0.85 |  |
| target | BMORI:acyl-coenzyme A dehydrogenase | HaOG201695 | CGCCAAACCAGAGAACAAGC | CCAGCTGCGTACTTGTAACC | 0.78 |  |
| target | BMORI:putative fatty acyl-CoA reductase CG5065-like | HaOG214659 | GCGTCGAGAACAAAGTCACAC | ATAGCTTTCAGACGCTGGCT | 0.89 |  |
| target | BMORI:3-oxoacyl-[acyl-carrier-protein] reductase chloroplastic-like | HaOG211178 | ATACTCCGCATCGAAGGCTG | TCAGTTTTCACGGGTCCAGG | 0.97 |  |
| target | BMORI:elongation of very long chain fatty acids protein 2-like | HaOG206659 | AGGTGGTCACGGAACTTTGA | TCTTAACACTGGGGACCGCT | 1.04 |  |
| target | BMORI:acyl-CoA Delta(11) desaturase | HaOG215659 | CGGCCAGAATTCCCTTCTCC | CGTTGTGAGGGTCTGCATCT | 0.94 |  |
| target | BMORI:3-hydroxyacyl-CoA dehydrogenase | HaOG208751 | GCAAGGATACTCCCGGCTTT | GCATCGCCTCTCTCCAACAT | 0.74 |  |
| target | BMORI:probable peroxisomal acyl-coenzyme A oxidase 1-like | HaOG208389 | TGAAGCCTGATGCTGGTGAG | TCGAGGCTATGGCGATGAAC | 0.97 |  |
| control | elongation factor | Yan *et al.* (2013) | GAAGTCAAGTCCGTGGAGATG | GACCTGTGCTGTGAAGTCG | 0.88 |  |
| control | actin | Wang *et al.* (2013) | TCCAGCCCTCATTCTTGGGTAT | CAAGTCCTTACGGATGTCAACA | 0.87 |  |

**Table S5. GO-term enrichment analysis of genes up-regulated in long-distance phenotypes of *H. armigera*.**

| GO-ID | Term | Category | FDR | #Test | #Ref |
| --- | --- | --- | --- | --- | --- |
| GO:0006189 | 'de novo' IMP biosynthetic process | P | 4.41E-08 | 6 | 3 |
| GO:0055114 | oxidation-reduction process | P | 1.03E-05 | 28 | 974 |
| GO:0044282 | small molecule catabolic process | P | 1.71E-05 | 9 | 71 |
| GO:1901606 | alpha-amino acid catabolic process | P | 5.22E-04 | 6 | 35 |
| GO:0009072 | aromatic amino acid family metabolic process | P | 5.22E-04 | 5 | 18 |
| GO:0006564 | L-serine biosynthetic process | P | 8.05E-04 | 3 | 1 |
| GO:0006164 | purine nucleotide biosynthetic process | P | 5.42E-03 | 7 | 91 |
| GO:0016742 | hydroxymethyl-, formyl- and related transferase activity | F | 8.06E-03 | 3 | 5 |
| GO:0030170 | pyridoxal phosphate binding | F | 1.42E-02 | 5 | 45 |
| GO:0050662 | coenzyme binding | F | 1.57E-02 | 9 | 199 |
| GO:0006546 | glycine catabolic process | P | 1.90E-02 | 3 | 8 |
| GO:0006760 | folic acid-containing compound metabolic process | P | 2.38E-02 | 3 | 9 |
| GO:0016903 | oxidoreductase activity, acting on the aldehyde or oxo group of donors | F | 3.43E-02 | 5 | 58 |
| GO:0016823 | hydrolase activity, acting on acid carbon-carbon bonds, in ketonic substances | F | 3.43E-02 | 2 | 1 |
| GO:0035999 | tetrahydrofolate interconversion | P | 3.43E-02 | 2 | 1 |
| GO:0004488 | methylenetetrahydrofolate dehydrogenase (NADP+) activity | F | 3.43E-02 | 2 | 1 |
| GO:0042219 | cellular modified amino acid catabolic process | P | 3.43E-02 | 2 | 1 |
| GO:0006144 | purine nucleobase metabolic process | P | 4.82E-02 | 3 | 13 |

***GO, gene ontology; FDR, false discovery rate**
