# Supplementary material for: Genomewide transcriptional signatures of migratory flight activity in a globally invasive insect pest
Source: Mol Ecol. 2015 Sep 28;24(19):4901–11. doi: 10.1111/mec.13362 (PMC5102652; doi:10.1111/mec.13362)
Supplement: Supplementary file 3 [file MEC-24-4901-s003.docx]

A list of differentially expressed genes, corresponding annotations and fold changes for the China and Greece flight mill experiments.
